# Variation in plant responsiveness to defense elicitors caused by genotype and environment

**DOI:** 10.3389/fpls.2014.00349

**Published:** 2014-07-21

**Authors:** Toby J. A. Bruce

**Affiliations:** Department of Biological Chemistry and Crop Protection, Rothamsted ResearchHarpenden, UK

**Keywords:** induced defense, genotype, crop protection, epigenomics, insect-plant interactions

## Introduction

The need to develop novel crop protection treatments that can be used in agriculture has driven much research into induced plant defense and is used as a justification for it. Plant defense elicitors could provide novel agrochemicals to protect crops from pests and diseases. However, in order to achieve this, treatments have to give consistent, reliable reductions in pest infestation or pathogen infection levels (Stephen Skillman, Syngenta, *personal communication*). When moving beyond controlled laboratory conditions one issue encountered has been the high variability of the induced defense approach—sometimes an effect is observed and sometimes it is not (Anderson et al., 2006; Wu and Baldwin, 2010). Inducing plant defenses is complex because the effect is via the plant, dependent on plant genetics and physiology and can be altered by the environmental context. This contrasts with conventional pesticides that have a direct toxic effect on target organisms and therefore more predictable effects.

This short opinion article will consider the role of plant genotype, environment and the interaction between genotype and environment in causing variation in induced plant defense responses.

## Genotype

**Figure 1:**
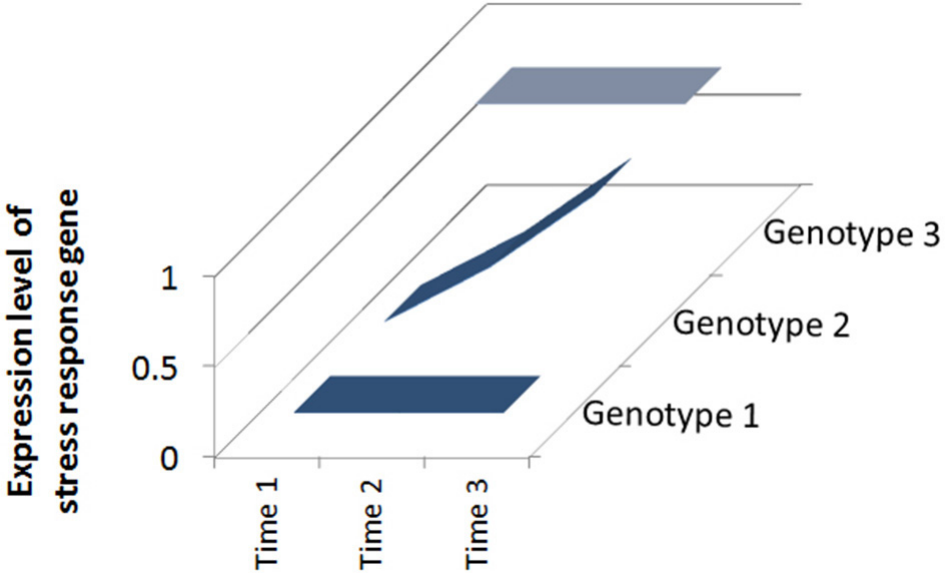
**Responsiveness of different plant genotypes to defense elicitors**. Over a period of stress (Time 1–Time 3), Genotype 1 has low initial and final expression levels of a stress response gene and is susceptible to attack; Genotype 2 has increasing expression levels because the gene is inducible and Genotype 3 has high initial and final expression levels because it is constitutively expressed.

Clearly not all plants are the same and some respond to defense elicitors better than others (Vallad and Goodman, 2004). The genotype of the plant can play a huge role in how well it responds to treatment. If particular inducible defense traits are absent in a given genotype nothing can be “switched on” by the treatment. Conversely, if a cultivar possesses inducible traits there is a “loaded gun” attached to the trigger (Figure 1). Many studies have revealed differences between plant genotypes in induced defense capacity and I will briefly review selected examples below.

There is natural variation between *Arabidopsis thaliana* accessions in resistance to *Botrytis cinerea* involving differences in *B. cinerea* induced camalexin accumulation and SA-dependent defenses (Denby et al., 2004; Rowe and Kliebenstein, 2008; Narusaka et al., 2013). Van Hulten et al. (2009) found natural variation in defense responsiveness amongst Arabidopsis accessions. Likewise, transcription profiling of wild Solanum species has revealed variation in induced defense responses between genotypes (Smith et al., 2014). Sharma et al. (2010) found that six tomato accessions varied significantly in inducibility of resistance against *Phytophthora infestans*. Natural variation in basal defense responsiveness to disease is reviewed by Ahmad et al. (2010).

Broekgaarden et al. (2007) found differences in transcriptional responses of two *Brassica oleracea* cultivars in response to induction by *Pieris rapae* attack. Of all the genes induced at any time point, only one third was induced in both cultivars tested. Similarly, Wu et al. (2008) found large differences between two *Nicotiana attenuata* accessions in signaling induced by oral secretions of the specialist herbivore *Manduca sexta*. Genotypic variation was observed in tomato when the cultivar “Carousel” responded to a seed treatment designed to induce defense whereas tomato cultivar “Moneymaker” did not (Smart et al., 2013). Some maize cultivars have a higher constitutive level of jasmonic acid (JA) based defenses as shown by Shivaji et al. (2010) which means there is less scope for further induction of them. The resistant inbred Mp708 had approximately 3-fold higher levels of jasmonic acid (JA) prior to herbivore feeding than the susceptible inbred Tx601. Han et al. (2009) found that wheat cultivars which were more resistant to aphids had greater constitutive levels of phenylalanine ammonia-lyase, polyphenol oxidase and peroxidase activity than susceptible ones. Aphid infestation also induced activity of these enzymes in all cultivars, especially in susceptible ones. However, resistant varieties sometimes have higher levels of both constitutive and induced defenses.

Herbivore induced volatile (HIPV) emission plays an important role in indirect defense whereby natural enemies are attracted to plants after exposure to insect attack. Schuman et al. (2009) found variation between accessions of *Nicotiana attenuata* in HIPV emission. Variation in (HIPV) emission between Arabidopsis accessions has been demonstrated and this influenced the behavior of the parasitoid *Diadegma semiclausum* when offered headspace volatiles in two-choice experiments (Snoeren et al., 2010). Certain maize lines respond to elicitors in stemborer eggs that induce HIPV emission to attract natural enemies of the herbivore but most commercial hybrid maize cultivars have lost this trait (Tamiru et al., 2011). Furthermore, Degen et al. (2012) found big differences in HIPV emission between six different maize lines and in a field trial there were significant differences between the lines in the numbers of *Spodoptera frugiperda* recovered from the plants, their average weight gain and parasitism rates. There is also variation among genetic lines of *Datura wrightii* in herbivore and methyl jasmonate-induced volatiles (Hare, 2007): volatile emission from some lines after insect damage or MeJA treatment was lower than from other lines even before damage or MeJA treatment. Kappers et al. (2011) found variation in HIPV emission between cucumber varieties after infestation of the plants with herbivorous spider mites (*Tetranychus urticae*) and this influenced the attraction of carnivorous natural enemies. They suggested that the foraging success of natural enemies of pests can be enhanced by breeding for crop varieties that release specific volatiles.

## Environment

The expression of induced plant defense responses is tightly regulated by the ecological context of the plant (Ballare, 2011). For a plant defense activator treatment to work well it must be well timed; timing is more critical than with a conventional pesticide. To induce or prime plant defenses, the treatment needs to be applied before the pest or disease attack as a preventative rather than curative treatment. However, a previous stress may have already switched on the defense and this may limit the magnitude of response to further elicitor treatment. Furthermore, plant responses to biotic stress are influenced by responses to abiotic stress (Suzuki et al., 2014).

The previous history of exposure can affect plant responsiveness. When *Pseudomonas putida* BTP1 infects roots of *Phaseolus vulgaris*, plants become more resistant to *Botrytis cinerea* on leaves (Ongena et al., 2005) and *Trichoderma asperellum* T203 root colonization of cucumber induces resistance to pathogens in above-ground parts of the plant (Shoresh et al., 2005). Infesting rice plants with the white-backed planthopper, *Sogatella furcifera*, dramatically increased the resistance of plants to rice blast, *Magnaporthe grisea* (Kanno and Fujita, 2003). Poelman et al. (2008) found that early season herbivory induces plant defense and differentially affects plant responses to subsequently colonizing herbivores. The specialist *Plutella xylostella* was more abundant on *Pieris rapae*-induced plants and preferred these plants over undamaged plants in oviposition experiments. This could perhaps be because the specialist is attracted to the HIPVs from its host plant. In contrast, the generalist *Mamestra brassicae* was more abundant on control plants and preferred undamaged plants for oviposition. The order of herbivore attack thus mediates the expression of plant defense phenotypes. There is negative crosstalk between plant defense pathways which means that attack by a different type of attacker could compromise responses to the defense elicitor (Bruce and Pickett, 2007). For example, attack that switches on the salicylic acid defense pathway would make a plant less responsive to a treatment designed to switch on the jasmonic acid defense pathway. For example, Zhang et al. (2009) showed that whiteflies interfere with indirect plant defense against spider mites in Lima bean.

## Interaction between genotype and environment

Plants are responsive to their environment and can adapt to stressful conditions. As described in the previous section, prior biotic or abiotic stress in the environment can alter how well a plant responds to subsequent treatment with a defense activator. Not only are these changes mediated by changes in metabolite levels and transcription factors but plants also have the capacity to reprogram expression levels of stress-response genes via epigenetic stress imprints (Bruce et al., 2007; Galis et al., 2009).

Evidence is accumulating that herbivore and pathogen attack can generate defense induction phenotypes across generations (Holeski et al., 2012; Kumar et al., 2013). Epigenetic changes can provide long lasting effects and even influence defense gene expression two generations later if the stress level is high enough (Luna et al., 2012). Dowen et al. (2012) profiled the DNA methylomes of Arabidopsis plants exposed to bacterial pathogen, avirulent bacteria, or salicylic acid (SA) and found numerous stress-induced differentially methylated regions, many of which were intimately associated with differentially expressed genes. The epigenomes of plants thus reflect the history of local genotype-environment interactions and much remains to be learnt about this. It is likely that epigenetic profiling can provide information about prior stress similar to how tree rings (Hughes and Brown, 1992) have been used to indicate previous drought stress. Although they would indicate more recent events, the level of detail about types of stress could be higher because of differential imprinting of different types of stress response genes.

## Conclusions

Reliable and predictable treatment effects are required for practical use of plant defense activators by growers and for commercialization of such crop protection products. However, the genotype of the plant, the environmental conditions and history of stress exposure, influence the magnitude of any boost in plant defenses obtained with an activator. Walters et al. (2013) have also highlighted host plant genotype and environmental considerations such as prior induction or trade offs between defense pathways as factors influence the field performance of induced resistance. It is not surprising that there is variation given that the defense activator treatment is only as good as the inducible plant defenses that it switches on.

Defense activators or elicitors need to be developed with the appropriate crop genotypes that can respond to the treatment. Variation between crop cultivars is a limitation if activators are developed ignoring this factor but it is also an opportunity to develop suitable packages of seeds and activator agrochemicals. Genetic variation in inducible defense traits complicates the use of plant defense activators but there is future potential to use particular plant activators in a package with selected crop cultivars that offer the best genetic potential for induced defense (Bruce, 2010; Kappers et al., 2011).

### Conflict of interest statement

The author declares that the research was conducted in the absence of any commercial or financial relationships that could be construed as a potential conflict of interest.
